# Notes and Notices

**Published:** 1896-11

**Authors:** 


### NOTES AND NOTICES.

THE STANDARD DICTIONARY.—Funk & Wagnalls Company, New York, have just received a single order from one firm for 100,000 copies of their celebrated *Standard Dictionary of the English Language*, amounting at retail to nearly one and a quarter millions of dollars. This is the largest single sale of so large a work ever made in America. Previous to this one large transaction, over 100,000 copies had been issued, and the company is still receiving many large orders from its subscription agents throughout the world.

THE FIFTIETH ANNIVERSARY NUMBER of the *Scientific American*, New York, is a really handsome and valuable publication of 72 pages. It reviews the progress of the past fifty years in the various sciences and industrial arts; and the various articles by the best scientific writers of the day are racily written and richly illustrated. The editors have accomplished the difficult task of presenting a compendium of information that shall be at once historical, technical, and popular. The interest never flags for a moment, and the story of the half century's growth is in itself a veritable compendium of valuable scientific information for future reference. Price, 10 cents per copy.

COMMERCIAL TRAVELERS' FAIR.—No fair ever held in America's famous mammoth amusement palace, the Madison Square Garden, New York, has been founded upon the broad and thorough organization work planned by its Director General, Col. A. B. de Frece, for the coming Commercial Travelers' Fair, which opens for a two weeks' run December 15th in the Garden.

The fair's aim is to raise $150,000 with which to complete the National Commercial Travelers' Home at Binghamton, N. Y.

Special Commissioners are being appointed for every State in the Union, and for England, France, Belgium, Germany, Austria, and Mexico, and are working for what will really be a Commercial Travelers' International Exposition. For Philadelphia and the manufacturing and commercial territory tributary to it, Mr. J. Harrington, lately of the Republican National Committee, and Mr. G. E. Martyn, of the Republican County Committee of New York, have been appointed Special Commissioners. The Commissioners will endeavor to interest all of Philadelphia's business men in supporting the fair through subscriptions, donations, advertising, and the purchase of season tickets, the holders of which will be awarded the valuable goods donated to the fair.

They will also represent Chairman Allen S. Williams, of the Commercial Travelers' Day Committee, in securing from everybody pledges of cash donations to mark December 1st, Commercial Travelers' Day.

Dr. C. E. Fisher, of the *Medical Century*, will return to this city and resume charge of the *Medical Century*, December 10th, 1896, having had a very beneficial outing on the Pacific Coast.

"Annales d'Occulistique."

33 West Thirty-Third Street, New York,
November 6th, 1896.

Dear Doctor:—In January, 1895, in the belief that the *Annales d'Occulistique*, through a provisional agreement prepared by the editors of that journal, was to be edited as a periodical in two editions, editions in which there was to be a unity of interest, I undertook, at an expense of much labor and means, the direction of the edition in English.

Interpretations were soon placed upon the agreement which I could not possibly accept nor even consider. These interpretations were such as to be absolutely incompatible with any unity of action or interest and fatal to the hope of any fulfillment of the promise to make the journal international.

After much delay, and with earnest efforts in the hope of a better outcome, I am compelled, with sincere regret, to announce that, in justice to the subscribers to the edition in English, to its contributors, and to myself, I have declined to continue to conduct it under existing conditions.

Seven numbers of the current year have been issued, including one full volume. Inclosed you will find a check for the amount of your subscription less the proportion for the completed volume.

I am very truly yours,

Geo. T. Stevens, M. D.

Detection of Sugar in the Urine.—Dr. A. R. Elliott gives the following formula devised by himself for the testing of sugar in the urine:

Cupric Sulphate, gr. xxvij.

Glycerine (pure), ʒ iij.

Distilled water, ʒ iiss.

Liq. Potassæ, ad. oz. iv.

Dissolve the Cupric Sulphate in the Glycerine and water, and gently heat. When cold add the Liquor Potassæ.

The "Standard Dictionary" of Funk & Wagnall's, so frequently and uniformly recommended by this journal, has among other excellent things a special form of grouping that seems to be original with the *Standard*—the gathering up under a particular subject of the principal technical words employed in it, so that if the reader requires a word and cannot recall it, he has but to turn to the branch to which it belongs, and the chances are that he will find a list of the principal terms used in the trade or business. Under *explosive*, *brewing*, *brick-making*, *printing*, *agriculture*, the various games, etc., are examples of this kind.

Still other forms of grouping are those by which the derivations of a word are run in under the main word (as *adventurish*, *adventurism*, *adventuresome*, *adventuresomeness* under *adventure*), and compounds and terms belonging to a particular subject are run in under it, sometimes without definition when self-explaining, with a word or two of parenthetical explanation when necessary. It is by such means as this that the editors of the *Standard* have not only been able to crowd so many words into such a small space, but at the same time to give information in bulk at the point naturally first consulted.

Fought for Triplets.—The wife of Joseph Silverstein gave birth to triplets on October 8th, and in the excitement two doctors were summoned. They both fought in the hall, and finally the police surgeon was called in. He chased the wrangling physicians away and rendered medical attendance. The mother is doing well.

Self-Protection in Boston.—A man of genteel breeding and intellectual force told us the other day that he wears sewed to his undershirt a card with this inscription: " My appendix has been cut out." And he gave this reason for his action: " You see, these are the palmy knifing days of the surgeon. If a man falls in a fit or faints, or is disguised mentally by a drug, and is carried consequently to a hospital, the surgeon operates on him for appendicitis without delay."—*Boston Journal.*

The "Scientific American."—This unrivaled periodical, now in its fifty-first year, continues to maintain its high reputation for excellence, and enjoys the largest circulation ever attained by any scientific publication. Every number contains sixteen large pages, beautifully printed, elegantly illustrated; it presents in popular style a descriptive record of the most novel, interesting, and important advances in all the principal departments of science, and the useful arts, embracing Biology, Geology, Mineralogy, Natural History, Geography, Archæology, Astronomy, Chemistry, Electricity, Light, Heat, Mechanical Engineering, Steam and Railway Engineering, Mining, Ship Building, Marine Engineering, Photography, Technology, Manufacturing Industries, Sanitary Engineering, Agriculture, Horticulture, Domestic Economy, Biography, Medicine, etc. A vast amount of fresh and valuable information pertaining to these and allied subjects is given, the whole profusely illustrated with engravings.

